# Supplementary material for: Authentication of Laying Hen Housing Systems Based on Egg Yolk Using 1H NMR Spectroscopy and Machine Learning
Source: Foods. 2024 Apr 3;13(7):1098. doi: 10.3390/foods13071098 (PMC11011716; doi:10.3390/foods13071098)
Supplement: Supplementary file 1 [file foods-13-01098-s001.zip › foods-2931181-supplementary.pdf]

Article

Authentication of Laying Hen Housing Systems Based on Egg Yolk Using 1H NMR Spectroscopy and Machine Learning

Greta Bischof [1,*], Edwin Januschewski [1] and Andreas Juadjur [1]

Chemical Analytics, German Institute of Food Technologies (DIL e.V.), Prof.-v.-Klitzing-Str. 7, 49610 Quakenbrück, Germany
* Correspondence: gretabischof@gmx.de

Supplemental material

Equations

Accuracy:

$$accuracy = \frac{TP + TN}{TP + TN + FP + FN} \tag{S1}$$

Sensitivity:

$$sensivity = \frac{TP}{TP + FN} \tag{S2}$$

Specificity:

$$specificity = \frac{TN}{TN + FP} \tag{S3}$$

Precision:

$$precision = \frac{TP}{TP + FP} \tag{S4}$$

Number of misclassification (NMC):

$$NMC = FP + FN \tag{S5}$$

Receiver Operating Characteristic Curve (ROC) and area under the curve (AUC): plot of sensitivity / 1-specificity at various thresholds.

Confusion matrix:

	Predicted Class 1	Predicted Class 2
True Class 1	True positive (TP)	False negative (FN)
True Class 2	False positive (FP)	True negative (TN)

Table S1. Samples of data set for model development. A total of 472 eggs were collected from colony cages, 1,200 eggs from barn, 1,192 eggs from free-range and 1,324 eggs from organic housing systems, resulting in a total amount of 4,188 eggs used for model development.

Samples	Housing system	Breed[1]	Age of hens [week]	Number of eggs per sample
**B-001**	Barn	LSL	22	72
**B-002**	Barn	Dekalb	75	63
**B-003**	Barn	Dekalb	33	64
**B-004**	Barn	LSL	32	74
**B-005**	Barn	Dekalb	23	59
**B-006**	Barn	LB	67	56
**B-007**	Barn	LB	28	70
**B-008**	Barn	Dekalb	58	71
**B-009**	Barn	Dekalb	39	75
**B-010**	Barn	LSL	66	55
**B-011**	Barn	LSL	51	45
**B-012**	Barn	LB	64	83
**B-013**[a]	Barn	LB	22	66
**B-014**[a]	Barn	LB	28	80
**B-015**[a]	Barn	LB	43	81
**B-016**[a]	Barn	LB	59	95
**B-017**	Barn	LSL	24	91
**K-001**	Colony cage	LSL	47	77
**K-002**	Colony cage	LSL	30	65
**K-003**	Colony cage	LSL	77	59
**K-004**	Colony cage	LSL	45	28
**K-005**	Colony cage	LSL	72	62
**K-006**	Colony cage	LSL	20	41
**K-007**	Colony cage	LSL	62	64
**K-008**	Colony cage	LSL	65	76
**F-001**	Free-range	LSL	33	69
**F-002**	Free-range	Dekalb	40	69
**F-003**	Free-range	Dekalb	53	64
**F-004**	Free-range	LB	60	77
**F-005**	Free-range	Dekalb	21	75
**F-006**	Free-range	LB	21	77
**F-007**	Free-range	Dekalb	34	64
**F-008**	Free-range	LSL	50	77
**F-009**	Free-range	Sandys	74	76
**F-010**	Free-range	Dekalb	64	75
**F-011**	Free-range	LB	44	78
**F-012**	Free-range	LSL	60	79
**F-013**	Free-range	LB	31	82
**F-014**	Free-range	Sandys	23	81
**F-015**	Free-range	Sandys	30	77
**F-016**	Free-range	Sandys	40	72
**O-001**	Organic	LB	41	79
**O-002**	Organic	LB	31	78
**O-003**	Organic	Dekalb	81	114
**O-004**	Organic	LSL	22	67
**O-005**	Organic	Sandys	29	78
**O-006**	Organic	LB	94	71
**O-007**	Organic	LSL	45	74

Samples	Housing system	Breed[1]	Age of hens [week]	Number of eggs per sample
**O-008**	Organic	Sandys	49	82
**O-009**	Organic	LB	24	66
**O-010**	Organic	Sandys	58	69
**O-011**	Organic	Dekalb	29	73
**O-012**	Organic	Dekalb	21	83
**O-013**	Organic	Sandys	65	69
**O-014**	Organic	LSL	61	78
**O-015**	Organic	Dekalb	37	75
**O-016**	Organic	Sandys	35	81
**O-017**	Organic	LSL	33	87

LSL – Lohmann Selected Leghorn; LB – Lohmann Brown

[1] same flock of laying hens

Table S2. Sample set purchased in supermarket.

Samples	Housing system as labeled*	color of egg shell	egg size
**V-001**	Organic	Brown	M
**V-002**	Free-range	Brown	M
**V-003**	Organic	Brown	-
**V-004**	Free-range	White	-
**V-005**	Organic	White	S
**V-006**	Barn	White	M
**V-007**	Organic	Brown	M
**V-008**	Free-range	Brown	L
**V-009**	Barn	Brown	L
**V-010**	Barn	Brown	M
**V-011**	Organic	Brown	-
**V-012**	Organic	Brown	M
**V-013**	Free-range	White	M
**V-014**	Barn	White	M
**V-015**	Free-range	White	M
**V-016**	Barn	White	M
**V-017**	Organic	Brown	L
**V-018**	Organic	White	M
**V-019**	Free-range	White	M
**V-020**	Barn	White	M
**V-021**	Barn	Brown	M
**V-022**	Free-range	Brown	M
**V-023**	Free-range	Brown	L
**V-024**	Barn	White	M
**V-025**	Free-range	Brown	-
**V-026**	Organic	Brown	-
**V-027**	Free-range	Mixed	M
**V-028**	Barn	White	L

* 0 – organic; 1 – free-range; 2 – barn

Table S3. Prediction of the housing system of the samples from the supermarket using machine learning models.

Sample	Housing system	LDA	QDA	PLS-DA	SVM	RF	kNN	ANN
V-001-01	Organic	Organic	Organic	Organic	Organic	Organic	Organic	Organic
V-001-02	Organic	Organic	Organic	Organic	Organic	Organic	Organic	Organic
V-001-03	Organic	Organic	Organic	Organic	Organic	Organic	Organic	Organic
V-001-04	Organic	Organic	Organic	Organic	Organic	Organic	Organic	Organic
V-001-05	Organic	Organic	Organic	Organic	Organic	Organic	Organic	Organic
V-001-06	Organic	Organic	Organic	Free-range	Organic	Organic	Organic	Organic
V-001-07	Organic	Organic	Organic	Organic	Organic	Organic	Organic	Organic
V-001-08	Organic	Organic	Organic	Organic	Organic	Organic	Organic	Organic
V-001-09	Organic	Organic	Organic	Organic	Organic	Organic	Organic	Organic
V-001-10	Organic	Organic	Organic	Organic	Organic	Organic	Organic	Organic
V-001-11	Organic	Organic	Organic	Organic	Organic	Organic	Organic	Organic
V-001-12	Organic	Organic	Organic	Organic	Organic	Organic	Organic	Organic
V-002-01	Free-range	Free-range	Free-range	Barn	Free-range	Free-range	Free-range	Free-range
V-002-02	Free-range	Free-range	Free-range	Free-range	Free-range	Free-range	Free-range	Free-range
V-002-03	Free-range	Free-range	Free-range	Free-range	Free-range	Free-range	Free-range	Free-range
V-002-04	Free-range	Free-range	Free-range	Free-range	Free-range	Barn	Free-range	Free-range
V-002-05	Free-range	Free-range	Free-range	Free-range	Free-range	Free-range	Free-range	Free-range
V-002-06	Free-range	Free-range	Free-range	Barn	Free-range	Free-range	Free-range	Free-range
V-002-07	Free-range	Free-range	Free-range	Free-range	Free-range	Free-range	Free-range	Free-range
V-002-08	Free-range	Free-range	Free-range	Free-range	Free-range	Free-range	Free-range	Free-range
V-002-09	Free-range	Free-range	Free-range	Free-range	Free-range	Free-range	Free-range	Free-range
V-002-10	Free-range	Free-range	Free-range	Free-range	Free-range	Free-range	Free-range	Free-range
V-002-11	Free-range	Free-range	Free-range	Free-range	Free-range	Free-range	Free-range	Free-range
V-002-12	Free-range	Free-range	Free-range	Free-range	Free-range	Free-range	Free-range	Free-range
V-003-01	Organic	Organic	Organic	Barn	Organic	Barn	Barn	Free-range
V-003-02	Organic	Organic	Organic	Organic	Organic	Barn	Barn	Organic
V-003-03	Organic	Organic	Organic	Organic	Organic	Barn	Organic	Free-range
V-003-04	Organic	Organic	Organic	Organic	Organic	Barn	Organic	Organic
V-003-05	Organic	Organic	Organic	Organic	Organic	Barn	Barn	Organic
V-003-06	Organic	Organic	Organic	Organic	Organic	Barn	Barn	Organic
V-003-07	Organic	Organic	Organic	Barn	Organic	Barn	Barn	Organic
V-003-08	Organic	Barn	Organic	Organic	Organic	Organic	Organic	Organic
V-003-09	Organic	Organic	Organic	Organic	Organic	Barn	Barn	Organic
V-003-10	Organic	Organic	Organic	Organic	Organic	Barn	Organic	Organic
V-003-11	Organic	Organic	Organic	Organic	Organic	Organic	Organic	Organic
V-003-12	Organic	Organic	Organic	Barn	Organic	Barn	Barn	Organic
V-004-01	Free-range	Free-range	Free-range	Free-range	Free-range	Organic	Free-range	Free-range
V-004-02	Free-range	Free-range	Free-range	Free-range	Free-range	Free-range	Free-range	Free-range
V-004-03	Free-range	Free-range	Free-range	Free-range	Free-range	Organic	Organic	Free-range
V-004-04	Free-range	Free-range	Free-range	Free-range	Free-range	Free-range	Free-range	Free-range
V-004-05	Free-range	Free-range	Free-range	Free-range	Free-range	Organic	Free-range	Free-range
V-004-06	Free-range	Free-range	Free-range	Free-range	Free-range	Free-range	Free-range	Free-range
V-004-07	Free-range	Free-range	Free-range	Free-range	Free-range	Barn	Free-range	Free-range
V-004-08	Free-range	Free-range	Free-range	Free-range	Free-range	Organic	Organic	Free-range

Sample	Housing system	LDA	QDA	PLS-DA	SVM	RF	kNN	ANN
V-004-09	Free-range	Free-range	Free-range	Free-range	Free-range	Barn	Free-range	Free-range
V-004-10	Free-range	Free-range	Free-range	Free-range	Free-range	Free-range	Free-range	Free-range
V-005-01	Organic	Organic	Organic	Organic	Organic	Organic	Organic	Organic
V-005-02	Organic	Organic	Organic	Organic	Organic	Organic	Organic	Organic
V-005-03	Organic	Organic	Organic	Organic	Organic	Organic	Barn	Organic
V-005-04	Organic	Organic	Organic	Organic	Free-range	Organic	Barn	Free-range
V-005-05	Organic	Organic	Organic	Organic	Organic	Organic	Barn	Free-range
V-005-06	Organic	Free-range	Free-range	Organic	Free-range	Organic	Organic	Organic
V-005-07	Organic	Organic	Organic	Organic	Free-range	Organic	Barn	Organic
V-005-08	Organic	Organic	Organic	Organic	Organic	Organic	Organic	Organic
V-005-09	Organic	Organic	Organic	Organic	Organic	Organic	Organic	Organic
V-005-10	Organic	Organic	Free-range	Organic	Organic	Organic	Barn	Organic
V-006-01	Barn	Free-range	Free-range	Barn	Free-range	Organic	Organic	Barn
V-006-02	Barn	Free-range	Free-range	Free-range	Free-range	Free-range	Free-range	Free-range
V-006-03	Barn	Free-range	Free-range	Barn	Free-range	Organic	Free-range	Free-range
V-006-04	Barn	Free-range	Free-range	Barn	Free-range	Free-range	Free-range	Free-range
V-006-05	Barn	Free-range	Free-range	Barn	Free-range	Organic	Barn	Free-range
V-006-06	Barn	Free-range	Free-range	Barn	Free-range	Organic	Barn	Free-range
V-006-07	Barn	Free-range	Free-range	Barn	Free-range	Organic	Barn	Free-range
V-006-08	Barn	Free-range	Free-range	Barn	Free-range	Organic	Barn	Free-range
V-006-09	Barn	Free-range	Free-range	Barn	Free-range	Organic	Barn	Barn
V-006-10	Barn	Free-range	Free-range	Free-range	Free-range	Organic	Barn	Free-range
V-007-01	Organic	Organic	Organic	Organic	Organic	Organic	Organic	Organic
V-007-02	Organic	Organic	Organic	Barn	Organic	Organic	Barn	Organic
V-007-03	Organic	Organic	Organic	Organic	Organic	Organic	Barn	Organic
V-007-04	Organic	Organic	Organic	Organic	Organic	Free-range	Free-range	Organic
V-007-05	Organic	Organic	Organic	Organic	Organic	Barn	Barn	Organic
V-007-06	Organic	Organic	Organic	Barn	Organic	Organic	Barn	Organic
V-007-07	Organic	Organic	Organic	Organic	Organic	Barn	Barn	Organic
V-007-08	Organic	Organic	Organic	Barn	Organic	Organic	Barn	Colony cage
V-007-09	Organic	Organic	Organic	Organic	Organic	Organic	Barn	Organic
V-007-10	Organic	Organic	Organic	Organic	Organic	Organic	Organic	Organic
V-008-01	Free-range	Organic	Organic	Barn	Organic	Barn	Barn	Organic
V-008-02	Free-range	Organic	Organic	Barn	Organic	Free-range	Barn	Free-range
V-008-03	Free-range	Free-range	Organic	Barn	Organic	Free-range	Barn	Organic
V-008-04	Free-range	Free-range	Organic	Barn	Free-range	Organic	Barn	Free-range
V-008-05	Free-range	Organic	Organic	Barn	Organic	Free-range	Barn	Organic
V-008-06	Free-range	Free-range	Organic	Barn	Free-range	Free-range	Barn	Free-range
V-008-07	Free-range	Free-range	Organic	Barn	Free-range	Free-range	Barn	Free-range
V-008-08	Free-range	Organic	Organic	Organic	Organic	Organic	Free-range	Free-range
V-008-09	Free-range	Free-range	Organic	Barn	Free-range	Organic	Free-range	Free-range
V-008-10	Free-range	Free-range	Organic	Barn	Organic	Organic	Free-range	none
V-009-01	Barn	Barn	Organic	Barn	Organic	Barn	Free-range	Organic
V-009-02	Barn	Organic	Organic	Barn	Organic	Organic	Organic	Organic
V-009-03	Barn	Organic	Organic	Organic	Organic	Organic	Organic	Free-range
V-009-04	Barn	Free-range	Organic	Barn	Organic	Barn	Organic	Free-range

Sample	Housing system	LDA	QDA	PLS-DA	SVM	RF	kNN	ANN
V-009-05	Barn	Organic	Organic	Free-range	Organic	Barn	Organic	Free-range
V-009-06	Barn	Organic	Organic	Organic	Organic	Organic	Organic	Organic
V-009-07	Barn	Free-range	Organic	Organic	Free-range	Organic	Free-range	Free-range
V-009-08	Barn	Organic	Organic	Organic	Organic	Organic	Organic	Free-range
V-009-09	Barn	Organic	Organic	Free-range	Organic	Organic	Organic	Free-range
V-009-10	Barn	Free-range	Organic	Free-range	Organic	Organic	Free-range	Organic
V-010-01	Barn	Barn	Organic	Barn	Organic	Barn	Barn	Organic
V-010-02	Barn	Colony cage	Organic	Organic	Organic	Barn	Barn	Organic
V-010-03	Barn	Organic	Organic	Organic	Organic	Barn	Barn	Organic
V-010-04	Barn	Organic	Organic	Organic	Organic	Barn	Barn	Organic
V-010-05	Barn	Colony cage	Organic	Barn	Organic	Barn	Barn	Organic
V-010-06	Barn	Organic	Organic	Organic	Organic	Barn	Barn	Organic
V-010-07	Barn	Colony cage	Organic	Colony cage	Organic	Barn	Barn	Colony cage
V-010-08	Barn	Organic	Organic	Organic	Organic	Barn	Barn	Organic
V-010-09	Barn	Colony cage	Organic	Colony cage	Organic	Barn	Barn	Organic
V-010-10	Barn	Colony cage	Organic	Barn	Organic	Barn	Barn	Colony cage
V-011-01	Organic	Organic	Organic	Free-range	Organic	Organic	Organic	Free-range
V-011-02	Organic	Organic	Organic	Free-range	Organic	Organic	Organic	Free-range
V-011-03	Organic	Organic	Organic	Free-range	Organic	Barn	Barn	Free-range
V-011-04	Organic	Organic	Organic	Free-range	Organic	Organic	Barn	Free-range
V-011-05	Organic	Organic	Organic	Free-range	Organic	Barn	Organic	Free-range
V-011-06	Organic	Organic	Organic	Free-range	Organic	Organic	Organic	Organic
V-011-07	Organic	Organic	Organic	Free-range	Organic	Organic	Free-range	Free-range
V-011-08	Organic	Organic	Organic	Free-range	Organic	Organic	Free-range	Organic
V-011-09	Organic	Organic	Organic	Free-range	Organic	Organic	Free-range	Free-range
V-011-10	Organic	Organic	Organic	Free-range	Organic	Organic	Free-range	Free-range
V-011-11	Organic	Organic	Organic	Free-range	Organic	Organic	Free-range	Free-range
V-011-12	Organic	Organic	Organic	Free-range	Organic	Organic	Free-range	Free-range
V-012-01	Organic	Organic	Organic	Free-range	Organic	Free-range	Free-range	Free-range
V-012-02	Organic	Organic	Organic	Free-range	Organic	Barn	Organic	Free-range
V-012-03	Organic	Organic	Organic	Free-range	Organic	Free-range	Free-range	Organic
V-012-04	Organic	Organic	Organic	Organic	Organic	Barn	Free-range	Organic
V-012-05	Organic	Organic	Organic	Free-range	Organic	Barn	Organic	Organic
V-012-06	Organic	Free-range	Free-range	Barn	Organic	Free-range	Free-range	Organic
V-012-07	Organic	Organic	Organic	Organic	Organic	Free-range	Free-range	Organic
V-012-08	Organic	Organic	Organic	Organic	Organic	Organic	Barn	Organic
V-012-09	Organic	Free-range	Organic	Free-range	Organic	Free-range	Organic	Free-range
V-012-10	Organic	Organic	Organic	Organic	Organic	Free-range	Free-range	Organic
V-012-11	Organic	Organic	Organic	Free-range	Organic	Organic	Organic	Free-range
V-012-12	Organic	Organic	Organic	Organic	Organic	Free-range	Barn	Free-range
V-013-01	Free-range	Free-range	Free-range	Free-range	Free-range	Free-range	Free-range	Free-range
V-013-02	Free-range	Free-range	Free-range	Free-range	Free-range	Free-range	Free-range	Free-range
V-013-03	Free-range	Free-range	Free-range	Free-range	Free-range	Free-range	Free-range	Free-range
V-013-04	Free-range	Free-range	Free-range	Free-range	Free-range	Free-range	Free-range	Free-range
V-013-05	Free-range	Free-range	Free-range	Free-range	Free-range	Free-range	Free-range	Free-range
V-013-06	Free-range	Free-range	Free-range	Free-range	Free-range	Organic	Free-range	Free-range

Sample	Housing system	LDA	QDA	PLS-DA	SVM	RF	kNN	ANN
V-013-07	Free-range	Free-range	Free-range	Free-range	Free-range	Free-range	Free-range	Free-range
V-013-08	Free-range	Free-range	Free-range	Free-range	Free-range	Free-range	Free-range	Free-range
V-013-09	Free-range	Free-range	Free-range	Free-range	Free-range	Free-range	Free-range	Free-range
V-013-10	Free-range	Free-range	Free-range	Free-range	Free-range	Free-range	Free-range	Free-range
V-013-11	Free-range	Free-range	Free-range	Free-range	Free-range	Free-range	Free-range	Free-range
V-013-12	Free-range	Free-range	Free-range	Free-range	Free-range	Free-range	Free-range	Free-range
V-014-01	Barn	Free-range	Organic	Free-range	Free-range	Free-range	Organic	Free-range
V-014-02	Barn	Free-range	Organic	Free-range	Free-range	Free-range	Free-range	Free-range
V-014-03	Barn	Free-range	Organic	Free-range	Free-range	Organic	Organic	Free-range
V-014-04	Barn	Free-range	Free-range	Barn	Free-range	Organic	Organic	Free-range
V-014-05	Barn	Free-range	Organic	Free-range	Free-range	Organic	Organic	Free-range
V-014-06	Barn	Free-range	Free-range	Free-range	Free-range	Free-range	Organic	Free-range
V-014-07	Barn	Free-range	Organic	Free-range	Free-range	Organic	Organic	Free-range
V-014-08	Barn	Free-range	Organic	Free-range	Free-range	Free-range	Organic	Free-range
V-014-09	Barn	Free-range	Organic	Free-range	Free-range	Organic	Organic	Free-range
V-014-10	Barn	Free-range	Free-range	Free-range	Free-range	Free-range	Free-range	Free-range
V-015-01	Free-range	Free-range	Organic	Barn	Organic	Organic	Organic	Free-range
V-015-02	Free-range	Free-range	Free-range	Free-range	Free-range	Barn	Organic	Free-range
V-015-03	Free-range	Free-range	Free-range	Free-range	Free-range	Barn	Free-range	Free-range
V-015-04	Free-range	Free-range	Free-range	Free-range	Free-range	Barn	Organic	Free-range
V-015-05	Free-range	Free-range	Free-range	Free-range	Free-range	Barn	Organic	Free-range
V-015-06	Free-range	Free-range	Free-range	Free-range	Free-range	Barn	Organic	Free-range
V-015-07	Free-range	Free-range	Barn	Free-range	Free-range	Barn	Organic	none
V-015-08	Free-range	Free-range	Organic	Free-range	Free-range	Organic	Organic	Free-range
V-015-09	Free-range	Free-range	Barn	Barn	Free-range	Barn	Organic	Free-range
V-016-01	Barn	Free-range	Organic	Free-range	Organic	Barn	Organic	none
V-016-02	Barn	Free-range	Free-range	Organic	Free-range	Organic	Organic	none
V-016-03	Barn	Free-range	Free-range	Free-range	Free-range	Barn	Organic	Free-range
V-016-04	Barn	Free-range	Free-range	Free-range	Free-range	Organic	Organic	Free-range
V-016-05	Barn	Free-range	Barn	Free-range	Free-range	Barn	Organic	Free-range
V-016-06	Barn	Free-range	Free-range	Free-range	Free-range	Barn	Organic	Free-range
V-016-07	Barn	Free-range	Free-range	Barn	Free-range	Barn	Organic	Free-range
V-016-08	Barn	Barn	Free-range	Barn	Free-range	Barn	Organic	Barn
V-016-09	Barn	Free-range	Free-range	Organic	Free-range	Organic	Organic	Free-range
V-016-10	Barn	Free-range	Free-range	Barn	Free-range	Barn	Organic	Free-range
V-017-01	Organic	Organic	Organic	Organic	Organic	Barn	Barn	Organic
V-017-02	Organic	Organic	Organic	Organic	Organic	Barn	Organic	Organic
V-017-03	Organic	Organic	Organic	Organic	Organic	Barn	Organic	Organic
V-017-04	Organic	Organic	Organic	Organic	Organic	Barn	Barn	Organic
V-017-05	Organic	Organic	Organic	Organic	Organic	Barn	Barn	Organic
V-017-06	Organic	Organic	Organic	Organic	Organic	Organic	Organic	Organic
V-017-07	Organic	Organic	Organic	Organic	Organic	Organic	Organic	Organic
V-017-08	Organic	Organic	Organic	Organic	Organic	Organic	Organic	Organic
V-017-09	Organic	Organic	Organic	Organic	Organic	Organic	Organic	Organic
V-018-01	Organic	Organic	Organic	Organic	Organic	Organic	Organic	Organic
V-018-02	Organic	Free-range	Organic	Organic	Organic	Organic	Organic	Organic

Sample	Housing system	LDA	QDA	PLS-DA	SVM	RF	kNN	ANN
V-018-03	Organic	Organic	Organic	Organic	Organic	Organic	Organic	Organic
V-018-04	Organic	Organic	Organic	Organic	Organic	Organic	Organic	Organic
V-018-05	Organic	Organic	Organic	Organic	Organic	Organic	Organic	Organic
V-018-06	Organic	Organic	Organic	Organic	Organic	Organic	Organic	Organic
V-018-07	Organic	Organic	Organic	Organic	Organic	Organic	Organic	Organic
V-018-08	Organic	Organic	Organic	Organic	Organic	Organic	Organic	Organic
V-018-09	Organic	Organic	Organic	Organic	Organic	Barn	Organic	Organic
V-019-01	Free-range	Barn	Free-range	Barn	Organic	Barn	Free-range	Free-range
V-019-02	Free-range	Barn	Organic	Barn	Organic	Barn	Organic	Barn
V-019-03	Free-range	Barn	Free-range	Barn	Organic	Barn	Barn	Barn
V-019-04	Free-range	Free-range	Free-range	Free-range	Organic	Organic	Free-range	Organic
V-019-05	Free-range	Organic	Free-range	Barn	Organic	Organic	Barn	Barn
V-019-06	Free-range	Barn	Free-range	Barn	Organic	Barn	Barn	Free-range
V-019-07	Free-range	Barn	Organic	Barn	Organic	Organic	Organic	Free-range
V-019-08	Free-range	Barn	Organic	Barn	Organic	Organic	Colony cage	Barn
V-019-09	Free-range	Barn	Organic	Barn	Organic	Organic	Free-range	Barn
V-020-01	Barn	Organic	Free-range	Organic	Organic	Organic	Free-range	Barn
V-020-02	Barn	Barn	Free-range	Organic	Organic	Organic	Free-range	Free-range
V-020-03	Barn	Barn	Free-range	Organic	Organic	Organic	Barn	Barn
V-020-04	Barn	Barn	Free-range	Barn	Organic	Organic	Free-range	Free-range
V-020-05	Barn	Barn	Free-range	Barn	Organic	Barn	Free-range	Free-range
V-020-06	Barn	Organic	Free-range	Organic	Organic	Organic	Organic	Free-range
V-020-07	Barn	Barn	Free-range	Organic	Organic	Barn	Free-range	Free-range
V-020-08	Barn	Barn	Organic	Organic	Organic	Barn	Barn	Barn
V-020-09	Barn	Organic	Free-range	Organic	Organic	Organic	Organic	Free-range
V-020-10	Barn	Barn	Free-range	Barn	Organic	Organic	Organic	Barn
V-021-01	Barn	Organic	Free-range	Organic	Organic	Barn	Barn	Free-range
V-021-02	Barn	Barn	Free-range	Barn	Organic	Barn	Barn	Free-range
V-021-03	Barn	Barn	Free-range	Barn	Organic	Barn	Barn	Barn
V-021-04	Barn	Barn	Free-range	Barn	Barn	Barn	Barn	none
V-021-05	Barn	Barn	Free-range	Barn	Organic	Barn	Barn	Barn
V-021-06	Barn	Barn	Free-range	Barn	Organic	Barn	Barn	Barn
V-021-07	Barn	Barn	Free-range	Barn	Barn	Barn	Barn	Barn
V-021-08	Barn	Barn	Free-range	Barn	Organic	Barn	Barn	Free-range
V-021-09	Barn	Barn	Free-range	Barn	Organic	Barn	Barn	Organic
V-021-10	Barn	Barn	Free-range	Barn	Organic	Barn	Barn	Organic
V-022-01	Free-range	Barn	Barn	Barn	Organic	Free-range	Barn	Free-range
V-022-02	Free-range	Barn	Free-range	Barn	Organic	Barn	Free-range	Barn
V-022-03	Free-range	Barn	Free-range	Barn	Organic	Barn	Free-range	Barn
V-022-04	Free-range	Barn	Free-range	Barn	Organic	Barn	Free-range	Organic
V-022-05	Free-range	Barn	Free-range	Barn	Organic	Free-range	Organic	Organic
V-022-06	Free-range	Organic	Free-range	Barn	Organic	Free-range	Free-range	Organic
V-022-07	Free-range	Organic	Free-range	Organic	Organic	Organic	Organic	Free-range
V-022-08	Free-range	Barn	Free-range	Barn	Organic	Free-range	Organic	Organic
V-022-09	Free-range	Barn	Barn	Barn	Organic	Free-range	Free-range	Free-range
V-022-10	Free-range	Organic	Organic	Organic	Organic	Barn	Organic	Organic

Sample	Housing system	LDA	QDA	PLS-DA	SVM	RF	kNN	ANN
V-023-01	Free-range	Organic	Organic	Organic	Organic	Organic	Free-range	Free-range
V-023-02	Free-range	Organic	Organic	Barn	Organic	Barn	Barn	Free-range
V-023-03	Free-range	Organic	Organic	Free-range	Organic	Barn	Barn	Free-range
V-023-04	Free-range	Free-range	Organic	Barn	Organic	Organic	Barn	Free-range
V-023-05	Free-range	Organic	Organic	Free-range	Organic	Organic	Free-range	Free-range
V-023-06	Free-range	Organic	Organic	Barn	Organic	Barn	Free-range	Organic
V-023-07	Free-range	Organic	Organic	Free-range	Organic	Organic	Organic	Free-range
V-023-08	Free-range	Organic	Organic	Organic	Organic	Barn	Organic	Free-range
V-023-09	Free-range	Organic	Organic	Free-range	Organic	Organic	Free-range	Free-range
V-023-10	Free-range	Organic	Organic	Barn	Organic	Organic	Barn	Free-range
V-024-01	Barn	Barn	Organic	Barn	Organic	Barn	Free-range	Barn
V-024-02	Barn	Barn	Free-range	Barn	Barn	Organic	Free-range	Barn
V-024-03	Barn	Barn	Organic	Barn	Organic	Barn	Free-range	Barn
V-024-04	Barn	Barn	Organic	Barn	Free-range	Barn	Free-range	Barn
V-024-05	Barn	Barn	Organic	Barn	Barn	Organic	Free-range	Barn
V-024-06	Barn	Barn	Organic	Barn	Organic	Organic	Free-range	Barn
V-024-07	Barn	Barn	Free-range	Barn	Organic	Barn	Free-range	Barn
V-024-08	Barn	Free-range	Organic	Free-range	Organic	Organic	Free-range	Free-range
V-024-09	Barn	Barn	Organic	Barn	Organic	Organic	Barn	Free-range
V-024-10	Barn	Barn	Organic	Free-range	Organic	Organic	Free-range	Free-range
V-025-01	Free-range	Organic	Free-range	Barn	Organic	Organic	Free-range	Organic
V-025-02	Free-range	Organic	Organic	Organic	Organic	Organic	Barn	Organic
V-025-03	Free-range	Barn	Free-range	Barn	Organic	Barn	Organic	Free-range
V-025-04	Free-range	Organic	Free-range	Organic	Organic	Organic	Organic	Organic
V-025-05	Free-range	Organic	Free-range	Organic	Organic	Barn	Organic	Organic
V-025-06	Free-range	Barn	Free-range	Organic	Organic	Organic	Organic	Free-range
V-025-07	Free-range	Barn	Free-range	Barn	Organic	Barn	Organic	Free-range
V-025-08	Free-range	Barn	Organic	Barn	Organic	Barn	Barn	Organic
V-025-09	Free-range	Barn	Free-range	Barn	Organic	Organic	Barn	Barn
V-025-10	Free-range	Free-range	Free-range	Organic	Organic	Organic	Organic	Free-range
V-026-01	Organic	Organic	Organic	Organic	Organic	Organic	Barn	Organic
V-026-02	Organic	Organic	Organic	Organic	Organic	Organic	Barn	Organic
V-026-03	Organic	Organic	Organic	Organic	Organic	Organic	Organic	Organic
V-026-04	Organic	Organic	Organic	Organic	Organic	Barn	Barn	Organic
V-026-05	Organic	Organic	Organic	Organic	Organic	Organic	Organic	Organic
V-026-06	Organic	Organic	Organic	Organic	Organic	Barn	Barn	Organic
V-026-07	Organic	Organic	Organic	Organic	Organic	Barn	Free-range	Organic
V-026-08	Organic	Organic	Organic	Organic	Organic	Organic	Organic	Organic
V-026-09	Organic	Organic	Organic	Barn	Organic	Organic	Organic	Colony cage
V-026-10	Organic	Organic	Organic	Organic	Organic	Barn	Organic	Organic
V-026-11	Organic	Organic	Organic	Organic	Organic	Organic	Barn	Organic
V-026-12	Organic	Organic	Organic	Organic	Organic	Barn	Barn	Organic
V-027-01	Free-range	Free-range	Free-range	Barn	Free-range	Organic	Organic	Free-range
V-027-02	Free-range	Free-range	Free-range	Organic	Free-range	Organic	Barn	Free-range
V-027-03	Free-range	Free-range	Free-range	Barn	Free-range	Barn	Free-range	Free-range
V-027-04	Free-range	Free-range	Free-range	Free-range	Free-range	Free-range	Organic	Free-range

Sample	Housing system	LDA	QDA	PLS-DA	SVM	RF	kNN	ANN
V-027-05	Free-range	Free-range	Free-range	Barn	Free-range	Free-range	Free-range	Organic
V-027-06	Free-range	Free-range	Free-range	Free-range	Free-range	Free-range	Organic	Free-range
V-027-07	Free-range	Free-range	Free-range	Barn	Free-range	Free-range	Free-range	Free-range
V-027-08	Free-range	Free-range	Free-range	Barn	Organic	Free-range	Barn	Free-range
V-027-09	Free-range	Free-range	Free-range	Free-range	Free-range	Free-range	Free-range	Free-range
V-027-10	Free-range	Organic	Free-range	Barn	Organic	Barn	Organic	Free-range
V-028-01	Barn	Free-range	Free-range	Free-range	Free-range	Barn	Organic	Free-range
V-028-02	Barn	Free-range	Free-range	Free-range	Free-range	Barn	Organic	Free-range
V-028-03	Barn	Free-range	Free-range	Free-range	Organic	Barn	Organic	none
V-028-04	Barn	Free-range	Free-range	Free-range	Free-range	Barn	Organic	Free-range
V-028-05	Barn	Free-range	Free-range	Barn	Free-range	Barn	Organic	Free-range
V-028-06	Barn	Free-range	Free-range	Barn	Free-range	Barn	Organic	Free-range
V-028-07	Barn	Free-range	Free-range	Free-range	Free-range	Free-range	Free-range	Free-range
V-028-08	Barn	Free-range	Free-range	Free-range	Free-range	Barn	Organic	Free-range
V-028-09	Barn	Free-range	Free-range	Free-range	Free-range	Organic	Organic	Free-range
V-028-10	Barn	Free-range	Free-range	Barn	Organic	Barn	Organic	Free-range
